# Supplementary material for: Psychometric performance of the CFQ-R-8D compared to the EQ-5D-3L and SF-6D in people with cystic fibrosis
Source: J Patient Rep Outcomes. 2024 Feb 28;8:24. doi: 10.1186/s41687-024-00697-w (PMC10900011; doi:10.1186/s41687-024-00697-w)
Supplement: Supplementary file 1 — Supplementary Material 1 [file 41687_2024_697_MOESM1_ESM.docx]

**Psychometric Performance of the CFQ-R-8D Compared to the EQ-5D-3L and SF-6D in People With Cystic Fibrosis**

**Abstract (288/350 words)**

**Objective:** This study aimed to compare the psychometric performance of the Cystic Fibrosis Questionnaire–Revised–8 Dimensions (CFQ-R-8D), a new, condition-specific, preference-based measure, with that of generic preference-based measures EQ-5D-3L and Short Form 6 dimensions (SF-6D).

**Methods:** Data from three trials of participants with CF aged ≥14 years who completed the CFQ-R and EQ-5D-3L or SF-6D were used. Analyses were undertaken to evaluate convergent validity based on correlations with CFQ-R domain scores. Known-group validity was assessed based on percent predicted forced expiratory volume in one second and pulmonary exacerbations. Responsiveness was based on correlation of change and sensitivity to change based on change in symptom severity. Effect sizes and standardized response means were estimated.

**Results:** CFQ-R-8D utilities and dimensions were strongly correlated with most of the overlapping CFQ-R domain scores (ρ>0.5); EQ-5D-3L and SF-6D utilities and dimensions had moderate (ρ>0.3) to strong correlations in dimensions capturing similar concepts. All measures showed evidence of known-group validity (*P*<0.05). Change correlations were strong for CFQ-R-8D utilities and dimensions and CFQ-R but they were moderate for SF-6D and mostly weak ((ρ>0.1) for EQ-5D-3L. The SF-6D had the largest mean change over time and effect sizes, followed by CFQ-R-8D and then EQ-5D-3L. Neither CFQ-R-8D or SF-6D utility scores had ceiling effects (<9% responses in full health) compared with those of EQ-5D-3L (61%-62%). In participants classified as being in full health by EQ-5D-3L, CFQ-R-8D captured CF-specific health problems, particularly cough, abdominal pain, and breathing difficulty.

**Conclusions:** The CFQ-R-8D reflected known-group differences and changes over time with stronger evidence of good psychometric performance than EQ-5D-3L and similar evidence as SF-6D. Additionally, the CFQ-R-8D captured more condition-specific symptoms than EQ-5D -3L or SF-6D, which are important determinants of health-related quality of life for people with CF.

**Keywords:** cost utility, cystic fibrosis, Cystic Fibrosis Questionnaire-Revised, Cystic Fibrosis Questionnaire–Revised–8 Dimensions, EQ-5D-3L, patient-reported outcomes, quality of life, Short Form 6 Dimensions; SF-6D, Short Form 6 dimensions

**Abbreviations:** CF, cystic fibrosis; CFQ-R, Cystic Fibrosis Questionnaire-Revised; CFQ-R-8D, Cystic Fibrosis Questionnaire–Revised–8 Dimensions; CFRSD, Cystic Fibrosis Respiratory Symptoms Diary; CFTR, cystic fibrosis transmembrane conductance regulator; HRQoL: health-related quality of life; NICE, National Institute for Health and Care Excellence; PEx, pulmonary exacerbation; ppFEV_1_; percent predicted forced expiratory volume in 1 second; QALY, quality-adjusted life year

**Background**

Economic evaluations support decision-making by assessing the costs and outcomes of different interventions. A common measure of outcomes is the quality-adjusted life-year (QALY), which combines length of life with utility values that reflect health-related quality of life (HRQoL) [1]. Utility values are often derived using generic preference-based measures of health such as the EQ-5D [2, 3], which are recommended by agencies such as the National Institute for Health and Care Excellence (NICE) in England and Wales [4]. Generic preference-based measures have been shown to be valid in many populations [5], but may miss important aspects of HRQoL in certain populations.

Cystic fibrosis (CF) is a genetic disorder caused by mutations in a gene that encodes a chloride-conducting transmembrane channel called the CF transmembrane conductance regulator (CFTR) [6]. CFTR dysfunction results in chronic infections and mucus retention followed by local airway inflammation that is harmful to the lungs. CF affects several body systems, but the largest impact is caused by progressive respiratory impairment. Evidence suggests that the EQ-5D-3L is not sensitive to meaningful differences in lung function (as measured by pulmonary function tests) and QoL (as measured by validated questionnaires) among people with CF [7]. A validated condition-specific measure, the CF Questionnaire–Revised (CFQ-R), covers general HRQoL dimensions (e.g., Physical Functioning, Vitality, Social, Role, and Emotional Functioning) as well as condition-specific dimensions (eg, Respiratory Symptoms, Body Image, Weight, Digestive Symptoms, Eating Disturbances, and Treatment Burden), and has been used to assess outcomes. However, the CFQ-R is not preference based and therefore cannot be used to derive health-state utilities. A new, condition-specific, preference-based measure, the CFQ–R–8 Dimensions (CFQ-R-8D), was developed from the CFQ-R to address these limitations [8].

Although the CFQ-R-8D is based on a well-validated measure, its psychometric performance has not yet been assessed relative to the longer, 50-item CFQ-R measure that it is drawn from or compared with the psychometric performance of generic preference-based measures in people with CF. Prior to use of any new measure, it is advisable to assess the psychometric performance of the measure, for example, to ensure that it is able to capture differences across different groups of severity and to capture changes over time due to therapy or disease progression. For preference-based measures that are used to generate utilities for use in health technology assessment by agencies such as NICE and the Pharmaceutical Benefits Advisory Committee, it is also recommended that research be conducted to understand how the utilities generated by the new measure differ from utilities generated by the generic preference-based measures [4, 9]. NICE, for example, recommends that psychometric evidence is provided to support the use of a condition-specific preference-based measure instead of EQ-5D, one of the most widely recommended measures [10], to generate QALYs in health technology assessment [4]. This can enable better understanding of how the utilities from the condition-specific preference-based measure differ from utilities from other measures and the potential impact on cost-effectiveness results.

The aim of this study was to compare the psychometric performance of the CFQ-R-8D with that of generic preference-based measures EQ-5D-3L and Short Form 6 dimensions (SF-6D) in people with CF and to assess differences in the utilities generated across the three measures.

**Methods**

Psychometric assessment of three measures, CFQ-R-8D, EQ-5D-3L, and SF-6D, was undertaken using data from three trials.

***Measures***

The condition-specific, preference-based CFQ-R-8D was developed from the CFQ-R adolescent/adult version, which has 50 questions for self-completion by those aged ≥14 years [11, 12]. Nine items from the CFQ-R were used to derive eight domains: Physical Functioning, Vitality, Emotion, Role Functioning, Cough, Breathing Difficulty, Abdominal Pain, and Body Image. The CFQ-R-8D was valued using time trade-off with a sample of the UK general population (n = 400) via face-to-face interviews. The health state utility values range from 0.236 to 1 [8].

Two generic preference-based measures were included: EQ-5D-3L and SF-6D. The EQ-5D-3L has five dimensions: mobility, self-care, usual activities, pain/discomfort, and anxiety/depression. EQ-5D-3L was scored using the UK tariff [2], which ranges from −0.594 to 1. The SF-6D is a classification system that was derived from the Short Form-12 [13]. Utility weights were generated using the UK tariff, which ranges from 0.345 to 1.

Other assessments were used to support the comparison of the preference-based measures, including the individual CFQ-R domain scores (range from 0 to 100, with higher scores indicating better QoL) and the CF Respiratory Symptom Diary (CFRSD), which focuses on pulmonary symptoms (difficulty breathing, tightness in chest, wheezing, coughing, fever, chills/sweats), emotional impacts (worry, sadness/depression, crankiness, frustration), and activity impacts (reduction of usual activities, work/study, tiredness, sleep, rest) [14]. CFRSD scores range from 0 to 100, with higher scores indicating more symptoms. Clinical assessments included percent predicted forced expiratory volume in 1 second (ppFEV_1_) and the number of pulmonary exacerbations (PEx) based on new or changed antibiotic therapy for four or more specified symptoms (eg, increased cough, increased dyspnea, and change in sputum).

***Data Sources***

Data were drawn from three trials. These included two phase 3, randomized, double-blind, placebo-controlled studies in which participants were randomly assigned to receive either lumacaftor (600 mg once daily or 400 mg every 12 hours) in combination with ivacaftor (250 mg every 12 hours) or matched placebo for 24 weeks (NCT01807923 and NCT01807949) [15]. These “EQ-5D Trials” included the adolescent and adult versions of the CFQ-R and EQ-5D-3L. Additional analyses were undertaken with data from a phase 3, randomized, double-blind, multicenter, placebo-controlled, parallel-group trial to evaluate combination therapy with tezacaftor (100 mg once daily) and ivacaftor (150 mg every 12 hours) (NCT02347657) [16]. This “SF-6D Trial” included the CFQ-R, CFRSD, and Short Form-12 version 2. For all three trials, participants who were aged ≥12 years, had CF, were homozygous for the *F508del-CFTR* mutation, had a ppFEV_1_ between 40% and 90% at screening, and had stable disease as judged by the investigator were eligible for inclusion.

Analyses reported here focus on data from the subgroup of participants aged ≥14 years because the CFQ-R-8D was derived from the adolescent and adult version of the CFQ-R, which was completed by this group. Younger participants completed different versions of the CFQ-R and the CFQ-R-8D could not be derived from these versions. The EQ-5D Trials sample included in this analysis (n = 1009) had a mean (SD) age of 26.2 (9.3) years, and 48.4% (n = 488) were female. The SF-6D Trial sample (n = 455) had a mean (SD) age of 27.8 (9.8) years, and 47.9% (n = 218) were female.

The data sets used for this validation study did not include treatment assignment variables, and all analyses were conducted using data pooled across treatment arms.

***Analysis***

To ensure comparison across measures for the same sample, the analysis used data at each time point from participants with complete responses for both CFQ-R-8D and either EQ-5D-3L or SF-6D and CFRSD, as none of the trials included all three measures. To ensure that we maximized the use of available data, other missing data (eg, in ppFEV_1_ and PEx) was not used to exclude participants, meaning that sample size differed across analyses. Baseline and follow-up means and SDs for each preference-based measure, the CFQ-R and CFRSD were estimated. Across all analyses, a nominal *P* value of 0.05 was considered statistically significant.

***Convergent Validity***

Based on COSMIN (COnsensus-based Standards for the selection of health Measurement INstruments) guidance, validity (i.e., the degree to which an instrument measures the construct it aims to measure) was assessed using convergent validity based on the relationship between the preference-based measures and the CFQ-R, which is a validated measure in this population. The CFRSD was also used in the SF-6D trial.

Pearson correlations were used for utility scores or total/dimension scores (CFQ-R and CFRSD) and Spearman rank correlations were used for dimensions. Correlations were assessed as: ≥0.5 as strong, <0.5 to ≥0.3 as moderate, and <0.3 as weak [17]. It was hypothesised that all the CFQ-R-8D dimensions would have strong correlations with the corresponding domains from the CFQ-R. It was expected that EQ-5D-3L would have weaker correlations than the CFQ-R-8D while strong correlations were expected for SF-6D dimensions that overlapped with the CFQ-R (physical functioning, mental health, pain and vitality). Utility scores are derived from members of the public and differ from scores such as those of the CFQ-R derived from people with CF – therefore hypotheses about how these would be related were based on differences across the measures rather than strength of correlation. The CFQ-R-8D utility scores were expected to be more strongly correlated to the CFQ-R domain scores than the EQ-5D-3L or the SF-6D utility scores. In the SF-6D trial, it was expected that the CFQ-R-8D dimensions and utilities would have moderate to strong correlations with the CFRSD, with strong correlations for dimensions related to pulmonary symptoms (Cough and Breathing Difficulty), emotional impacts (Emotional Functioning), and activity impacts (Physical and Role Functioning, and Vitality). SF-6D dimensions and utilities were expected to have smaller strength correlations with CFRSD compared to the CFQ-R-8D correlations. Convergent validity was evaluated separately at baseline and follow-up to assess whether convergence was similar at different time points, as clinical trial inclusion criteria may restrict the range of scores at baseline and thus may impact correlations.

***Known-Group Validity***

Validity can also be assessed based on the ability of measures to discriminate between known groups [18, 19]. CFQ-R-8D, EQ-5D-3L and SF-6D utility scores were assessed. CFQ-R did not have an overall score therefore it was not assessed. The CFRSD was assessed in the SF-6D trial as it measured CFQ related impacts. Known-groups were defined based on different levels of symptom severity for ppFEV_1_ using <40%, ≥40 to <70%, and ≥70% ppFEV_1_ cut-offs consistent with clinical trial reports [15] and the presence of less than 4 versus 4 or more PEx at 20 to 24 weeks. We also examined groups based on age at screening, comparing adolescents (aged 14 to <18 years) and adults (aged ≥18 years). Assessment was based on overall F test from an analysis of variance or *t* test and effect sizes (ie, the difference in mean scores between two adjacent subgroups divided by the pooled SD of scores). Effect sizes of ≥0.2 to <0.5, ≥0.5 to <0.8, and ≥0.8 denote small, medium, and large effect sizes, respectively [17]. The CFQ-R-8D was hypothesised to have larger effect sizes than the EQ-5D-3L and SF-6D but smaller effect sizes than the CFRSD.

***Responsiveness and sensitivity to change***

Responsiveness, the ability of an instrument to detect change over time in the construct that is being measured, was assessed using correlations in change between the CFQ-R and the three preference-based measures[18, 19]. In the trials, the primary target was a change in respiratory function and this was likely to result in change in the respiratory dimension and dimensions that would be affected by the physical and emotional impact of breathing (physical, role, emotional and social functioning, and vitality). Therefore, change correlations were expected to be strong between these CFQ-R domain scores and the CFQ-R-8D equivalent dimensions but would be moderate for SF-6D for overlapping dimensions and weak in the other dimensions. EQ-5D-3L has only 3 response levels in each of the five dimensions which may limit change and therefore change correlations were expected to be weak.

Sensitivity to change was also assessed based on groups that experienced change which were defined based on clinical expert judgement of meaningful ppFEV_1_ change over time (ie, improvement in ppFEV_1_ as ≥2 percentage points, no change from baseline as <2 to >−2 percentage points, and worsening as ≤−2 percentage points) or shift between severity groups (ie, <40%, ≥40 to 70%, and ≥70%) and PEx frequency (<4 and ≥4). Standardized response means (i.e., mean change score of a measure between two different time points divided by the SD of the change score) and change effect sizes were estimated. Effect size in this case was the mean change score of a measure between two different time points divided by the SD of the score at baseline. Either metric can be used to assess responsiveness; change effect size ignores variation in the change over time, which may be relevant for the current analysis as we did not include treatment group and thus, did not account for variability due to treatment effect [20]. Effect sizes and standardized response means of ≥0.2 to <0.5, ≥0.5 to <0.8, and ≥0.8, denote small, medium, and large effect sizes/standardized response means, respectively, which were numerically compared across the measures. CFQ-R-8D utility scores were hypothesised to have larger standardised response means and effect sizes for those who improved than SF-6D or EQ-5D-3L.

Floor (i.e., proportion at the worst health) and ceiling (i.e., proportion at the best health) effects were compared across the measures alongside distribution of the utilities for each measure, as these characteristics impact the ability to detect change. CFQ-R-8D utility scores were condition-specific and therefore were hypothesised to have lower ceiling effects than the EQ-5D-3L and the SF-6D.

***Ability to Capture Utility for CF-specific Health Problems***

Observed frequencies of each dimension of the CFQ-R-8D were reported when the generic preference-based measures were at full health to assess the ability of the CFQ-R-8D to detect a health deterioration at the ceiling of each generic preference-based measure. It was hypothesised that the CFQ-R-8D would be able to detect such health deterioration.

**Results**

Pooling across treatment arms, the mean (SD) CFQ-R-8D utility scores were 0.812 (0.12) and 0.803 (0.12) at baseline, and 0.816 (0.13) and 0.811 (0.13) at follow-up in the EQ-5D-3L and SF-6D trials, respectively (**Figure 1**). The mean (SD) EQ-5D-3L utility scores were 0.910 (0.13) at baseline and 0.909 (0.14) at follow-up in the EQ-5D-3L trials, while SF-6D utility scores were 0.802 (0.12) at baseline and 0.812 (0.13) at follow-up in the SF-6D trial. The CFQ-R summary scores also had similar mean scores and SDs at both baseline and follow-up within each domain (see **Supplementary Table 1**).

***Convergent Validity (CFQ-R-8D, EQ-5D-3L, SF-6D)***

As expected, all the CFQ-R-8D dimensions were strongly correlated with the overlapping domains in the CFQ-R (|0.64 to 0.82|). CFQ-R-8D utilities were also strongly correlated with CFQ-R dimension scores (|0.5 to 0.77|) with the exception of Body Image which had moderate correlations (|0.31 to 0.42|) (**Supplementary Tables 2**-**5**). CFQ-R-8D utility was also strongly correlated (ρ>0.5) with other CFQ-R domain scores, with the exception of Weight (ρ=0.20 at baseline), Eating Disturbance, and Treatment Burden (all moderate) (**Supplementary Tables 2-5**).

EQ-5D-3L dimensions (mobility, usual activities, pain or discomfort, and anxiety or depression) had weak to moderate correlations (|0.28 to 0.45|) with the CFQ-R domains where there was conceptual overlap while the utility scores were mostly moderate strength correlations (**Supplementary Table 2 and 3)**.

As expected, SF-6D dimensions that overlapped with CFQ-R dimensions had strong correlations (|0.59 to 0.76) and this was reflected in strong correlations with the SF-6D utilities (**Supplementary Table 4 and 5**). In the SF-6D trial, the CFQ-R-8D dimensions that overlapped with constructs in the CFRSD were strong (|0.51 to 0.67|) for Breathing Difficulty, Cough, and Vitality as expected but the correlations for Physical, Emotional and Role functioning ranged from strong to moderate (**Supplementary Table 4 and 5**). Similar correlations were found for the SF-6D with the CFRSD. CFQ-R-8D utility scores had stronger correlations with the CFRSD |0.67 to 0.7|) than the SF-6D (|0,50 to 0.55|).

***Known-Group Validity (CFQ-R-8D, EQ-5D-3L, SF-6D)***

All the preference-based measures demonstrated known-group validity. Across all data sets, there were statistically significant differences in CFQ-R-8D utilities by symptom severity measured using ppFEV_1_ at both time points and PEx at follow-up (**Tables 1 and 2**) with small to medium effect sizes. EQ-5D-3L utility scores were able to differentiate at follow-up for ppFEV_1_ and PEx with small effect sizes (**Table 1**).

SF-6D had statistically significant differences based on known groups with small to medium effect sizes. CFRSD also had statistically significant differences for known groups with small to medium effect sizes (**Table 2**). CFRSD had the largest effect sizes for symptom severity measured using ppFEV_1_, followed by CFQ-R-8D and SF-6D. Effect sizes were nearly identical for symptom severity measured using PEx across all measures, and all measures detected statistically significant differences in utilities between the adolescent (aged 14 to <18 years) and adult (aged ≥18 years) age groups.

***Responsiveness and sensitivity to change (CFQ-R-8D, EQ-5D-3L, SF-6D)***

Correlation between change in the CFQ-R-8D dimensions and the overlapping CFQ-R domains were strong as expected (|0.52 to 0.78|) while the correlations with the CFQ-R-8D utilities were moderate to strong for the overlapping domains but they were weak for the Body Image domain (**Supplementary Tables 6 and 7**).

EQ-5D-3L dimension and utilities had mostly weak correlations as expected (**Supplementary Table 6**) while SF-6D dimension and utilities had moderate to weak correlations with the CFQ-R domains where there was overlap (**Supplementary Table 7**).

Change correlations for the CFQ-R-8D Cough dimension were strong with change in CFRSD while the other overlapping constructs had moderate correlations while SF-6D change had smaller correlations (**Supplementary Table 7**).

Overall mean change between baseline and follow-up was very small (change effect size <0.2) for all measures, which was not unexpected, as approximately one-half of the patient sample was derived from placebo arms (**Supplementary Table 6**). When change was assessed based on changes in ppFEV_1_ and presence of PEx, change in utilities was in the expected direction for the CFQ-R-8D and the EQ-5D-3L, although the differences between groups by symptom severity were small (**Table 3**).

Standardized response means and effect sizes were small for CFQ-R-8D based on ppFEV_1_ change groups with the exception of standardized response means for the no change group (0.01), which was expected. In contrast, for EQ-5D-3L, standardized response means were mostly <0.2, which indicates little or no response. Both measures had similar negative changes in utility scores for those who had experienced four or more PEx; CFQ-R-8D showed a moderate effect size, while the EQ-5D-3L effect size was small due to greater variance in the EQ-5D-3L data.

Both CFQ-R-8D and SF-6D captured an increase in utility for participants showing an improvement in ppFEV_1_, but they did not reflect decreases in utility for those with ppFEV_1_ decline (**Table 3**). When change was assessed in movement across the ppFEV_1_ severity groups, SF-6D performed better than CFQ-R-8D, although most participants did not change in their severity group. The CFQ-R-8D and the SF-6D performed similarly for PEx, with both able to detect a utility reduction for participants with PEx and a small increase in utility for participants with no PEx. The effect sizes were small for both measures, although they were larger for CFQ-R-8D than SF-6D for participants who experienced an improvement in ppFEV_1_ (**Table 3**).

CFRSD was able to reflect changes based on change in ppFEV_1_ and PEx symptom severity group. Standardized response means and effect sizes for the CFRSD were larger or equivalent to those of the CFQ-R-8D and larger than those of the SF-6D for ppFEV_1_ changes but smaller on the other measures of change (**Table 3**).

The EQ-5D-3L had large ceiling effects at both baseline and follow-up across all dimensions (**Table 4**). The ceiling effects varied from 61% to 62% at baseline and follow-up for the utility score and from 81% to 99.7% by dimension. CFQ-R-8D and SF-6D did not have ceiling effects for the utility scores, but there was evidence of ceiling effects in some dimensions (**Table 4**). For CFQ-R-8D, Physical Functioning, Role Functioning, Abdominal Pain, and Body Image dimensions had approximately 60% to 75% responses at the ceiling. The other CFQ-R-8D dimensions had lower proportions at the ceiling, but only Cough was <10%. SF-6D dimensions showed a similar pattern, with ceiling effects ranging from 49% to 74% with the exception of energy (7% to 10%). CFRSD score did not suffer from ceiling effects. There were no individuals with the lowest scores across any of the measures. At the dimension level, there were no floor effects, with the exception of role limitation in SF-6D (23%).

***Ability to Capture Utility for Participants with CF-specific Health Problems (CFQ-R-8D, EQ-5D-3L, SF-6D)***

Mean (SD) CFQ-R-8D scores for those who reported a utility value of 1 for EQ-5D-3L (n = 607/997 [60.9%]) and SF-6D (n = 24/413 [5.8%]) were 0.866 (0.08 [range: 0.401-1]) and 0.916 (0.07 [range: 0.710-1]), respectively. When the EQ-5D-3L categorized participants as having no health problems, CFQ-R-8D was able to capture health problems, especially in Cough (n = 565/607 [93%]), but also in the CFQ-R-8D dimensions of Vitality, Emotion, Breathing Difficulty, and Abdominal Pain. There were fewer participants reporting no problems in SF-6D, but these participants had problems in CFQ-R-8D dimensions of Cough and Abdominal Pain (**Table 5**).

**Discussion**

This study assessed the psychometric performance of the newly developed condition-specific CFQ-R-8D in comparison with the generic EQ-5D-3L and SF-6D using existing trial data of participants with CF. Overall, CFQ-R-8D outperformed the EQ-5D-3L across all psychometric assessments and showed similar evidence of good psychometric performance as SF-6D.

CFQ-R-8D had better construct validity based on convergent validity in relation to cystic fibrosis health related quality of life – with strong correlations for dimensions and utility scores with the CFQ-R and the CFRSD as hypothesised, indicating construct validity. The only exception was for the Body Image domain from the CFQ-R which did not have strong correlations with the CFQ-R-8D utility score. As noted, utility scores come from members of the public; their preferences for Body Image were lower relative to the other dimensions of the CFQ-R-8D [8]. SF-6D had overlapping dimensions with the CFQ-R resulting in strong correlations whereas the correlations were of weaker strength for the EQ-5D-3L. As neither the EQ-5D-3L nor the SF-6D had respiratory related dimensions, they were not able to reflect this construct as well as the CFQ-R-8D.

The analyses reported evidence of known-group validity for CFQ-R-8D, EQ-5D-3L, and SF-6D, examined using symptom severity groups defined using ppFEV_1_ and PEx. Overall, CFQ-R-8D had stronger evidence of known-group validity than both EQ-5D-3L and SF-6D. However, the CFRSD had larger effect sizes than all the measures; this was not unexpected since it is a clinical measure that is focused on respiratory symptoms, and the known groups were defined based on symptom severity. CFQ-R-8D, EQ-5D-3L, and SF-6D all had higher utility scores for adolescents than for adults, which reflects the expected relationship between increased age and poorer health.

The correlation in changes in the three measures with the CFQ-R domain scores were as expected with strong correlations for the CFQ-R-8D dimensions, moderate correlations for the SF-6D dimensions, and weak to moderate correlations for the EQ-5D-3L. This supports the responsiveness of the CFQ-R-8D and that this was better than for the two other preference-based measures. Change in the CFQ-R-8D utility scores also had larger correlations (0.2 to 0.63) with change in CFQ-R domain scores compared to the EQ-5D-3L (0.08 to 0.33) or SF-6D (|0.06 to 0.43) utility scores. Therefore the CFQ-R-8D was evidence that the CFQ-R-8D utility scores were also more responsive than the two other preference-based measures.

All three measures showed some sensitivity to change. Mean change for participants who had improvements in ppFEV_1_ ranged from 0.019 to 0.035 in EQ-5D-3L and 0.024 to 0.041 in CFQ-R-8D, while this ranged from 0.026 to 0.056 in SF-6D. All measures also reflected the presence of PEx with negative mean change (−0.031 and −0.051 for CFQ-R-8D; −0.046 for EQ-5D-3L; and −0.039 for SF-6D). Both CFQ-R-8D and SF-6D had larger changes than the EQ-5D-3L. In addition, although there were changes in EQ-5D-3L score, it generally showed smaller standardized response means and effect sizes than CFQ-R-8D due to larger SDs indicating more variation. These larger SDs would be reflected in more uncertainty when EQ-5D-3L values are used in health technology assessment.

All measures exhibited ceiling effects across multiple dimensions, meaning that the measures cannot capture an improvement for participants who are already reporting no problems with the dimension. EQ-5D-3L had much larger ceiling effects than CFQ-R-8D and SF-6D and reported large ceiling effects for utility scores that were not observed for CFQ-R-8D or SF-6D. The high level of ceiling effects, with nearly two-thirds of patients at the maximum EQ-5D-3L value at baseline, calls into question the face validity of the use of EQ-5D-3L in people with CF. High ceiling effects may also have been a reflection of trial inclusion/exclusion criteria; however, enrolment criteria for the CF trials were broad and people with CF with lung function below the enrolment criteria (ie, ppFEV_1_ <40%) comprised a minority of the CF population. Moreover, the baseline EQ-5D-3L values for participants with CF in the studies were higher than those of UK population norms [21], which indicates that the severity of CF was not reflected by EQ-5D-3L. Future research could assess whether the 5-level version of EQ-5D, the EQ-5D-5L, has better performance.

Differences across classification systems were investigated, particularly for participants reporting full health in EQ-5D-3L and SF-6D. These differences indicated that the CFQ-R-8D was able to capture health problems, particularly cough, where EQ-5D-3L and SF-6D classify participants as being in full health. For the CF population, this is important because cough is a common symptom affecting HRQoL. This ability to capture health problems not captured by the EQ-5D-3L and SF-6D is a meaningful contribution of this condition-specific preference-based measure over the generic measures.

Overall, the selection of a preference-based measure that is used to generate QALYs is likely to impact the QALY results, and hence the incremental cost-effectiveness ratio. CFQ-R-8D demonstrated better construct validity and responsiveness than the other two measures. It was also able to demonstrate sensitivity to change. In general, EQ-5D-3L utilities are higher and show smaller differences across symptom severity groups and smaller change over time. In contrast, SF-6D and CFQ-R-8D utilities have similar values and similar differences across symptom severity groups and changes over time, with CFQ-R-8D sometimes having larger differences. This suggests that utility values generated using CFQ-R-8D would be most similar to SF-6D utilities. At the participant level, the CFQ-R-8D would be expected to capture more condition-specific symptoms—cough in particular, followed by vitality, abdominal pain, and breathing difficulty. Other studies that have assessed the performance of condition-specific and generic preference-based measures found that condition-specific measures may improve performance compared with EQ-5D-3L for milder health states, as condition-specific measures are not prone to ceiling effects and they target relevant symptoms [22]. Some studies have found mean change in EQ-5D-3L score to be larger than mean change in condition-specific, preference-based measures [22]; however, this was not the case in this study. An analysis using the CFQ-R-8D to calculate utility values from clinical trial data demonstrated a utility benefit of 0.085 for participants treated with the CF treatment elexacaftor/tezacaftor/ivacaftor vs those treated with placebo, when controlling for post-treatment lung function (Data on file: REF-19105). A similar benefit was estimated using change from baseline with real-world elexacaftor/tezacaftor/ivacaftor treatment in several countries (utility increase of 0.089, controlling for increase in lung function; Data on file REF- 22775). These applications suggest that the CFQ-R-8D may be a reliable and useful measure in evaluating the utility benefit of treatments for CF.

Limitations of this study included that the analyses were conducted on trial data that were also used to develop the classification system for CFQ-R-8D. This may have had some impact on the psychometric performance of the CFQ-R-8D since the items for the classification were, in part, selected based on the psychometric performance in these data sets. In addition, the relatively high CFQ-R scores at baseline may indicate that these trial populations did not cover the range of symptom severity for people with CF. Most participants had normal or mild symptom severity based on ppFEV_1_. Partly due to this, assessment of change over time was based on broad groups, particularly for changes in severity category for ppFEV_1_, which may mask differences. The assessments were also based on pooled data over treatment arms which may limit the responsiveness assessment of the CFQ-R-8D, although the measure was able to reflect differences based on other measures of severity over time. Therefore, assessment of the psychometric performance of CFQ-R-8D in other data sets of people with CF is recommended. Any comparisons of preference-based measures must also take into account the different sources of utility values for the measures: time trade-off for CFQ-R-8D and EQ-5D-3L and standard gamble for SF-6D using different protocols. The EQ-5D-3L and SF-6D valuation studies were conducted ≥17 years ago, whereas the CFQ-R-8D valuation study was conducted in 2019, and general population preferences may have changed over this time [2, 13]. It is not possible to account for these differences, but it is worth noting that they may have had an impact.

Furthermore, the different measures have different recall periods for people completing the measure. EQ-5D-3L asks people to report their health today, whereas CFQ-R (and hence CFQ-R-8D) asks people to report their health over the last 2 weeks, and SF-6D asks people to report their health over the last 4 weeks. These different recall periods could have impacted the results because participants’ health may have differed across the different recall periods.

**Conclusions**

Despite these limitations, the analysis presented here provides a good evidence base for the performance of CFQ-R-8D in people with CF relative to two commonly used generic preference-based measures across different trials. The CFQ-R-8D showed stronger evidence of good psychometric performance than EQ-5D-3L and similar evidence as SF-6D. In addition, the CFQ-R-8D captured more condition-specific symptoms than EQ-5D-3L or SF-6D, which are important determinants of HRQoL for people with CF.

**Declarations**

***Ethics Approval and Consent to Participate*:** For all of the studies contributing data, an ethics committee at each of the trial sites reviewed and approved the protocols. All patients or their parent/legal guardian provided written informed consent to participate in those studies and for the use of the study data for research.

***Consent for Publication*:** Not applicable

***Availability of Data and Materials*:** Included in the Title page submission. Left out of this document to ensure double-blinded peer review.

***Competing Interests*:** Included in the Title page submission. Left out of this document to ensure double-blinded peer review.

***Funding*:** Included in the Title page submission. Left out of this document to ensure double-blinded peer review.

***Authors’ Contributions*:** Included in the Title page submission. Left out of this document to ensure double-blinded peer review.

***Acknowledgments*:** Included in the Title page submission. Left out of this document to ensure double-blinded peer review.

**References**

[1] Prieto L, Sacristan JA. Problems and solutions in calculating quality-adjusted life years (QALYs). Health Qual Life Outcomes. 2003;1:80.

[2] Dolan P. Modeling valuations for EuroQol health states. Med Care. 1997;35:1095-108.

[3] EuroQol Research Foundation. EQ-5D-3L user guide. Rotterdam, the Netherlands: EuroQol Research Foundation; 2018.

[4] National Institute for Health and Care Excellence. Guide to the methods of technology appraisal 2013. 2013.

[5] Finch AP, Brazier JE, Mukuria C. What is the evidence for the performance of generic preference-based measures? A systematic overview of reviews. Eur J Health Econ. 2018;19:557-70.

[6] Elborn JS. Cystic fibrosis. Lancet. 2016;388:2519-31.

[7] Solem CT, Vera-Llonch M, Liu S, Botteman M, Castiglione B. Impact of pulmonary exacerbations and lung function on generic health-related quality of life in patients with cystic fibrosis. Health Qual Life Outcomes. 2016;14:63.

[8] Acaster S, Mukuria C, Rowen D, Brazier JE, Wainwright CE, Quon BS, et al. Development of the Cystic Fibrosis Questionnaire-Revised-8 Dimensions: estimating utilities from the Cystic Fibrosis Questionnaire-Revised. Value Health. 2023.

[9] The Pharmaceutical Benefits Advisory Committee. Guidelines for preparing a submission to the Pharmaceutical Benefits Advisory Committee version 5.0. The Pharmaceutical Benefits Advisory Committee; 2016.

[10] Kennedy-Martin M, Slaap B, Herdman M, van Reenen M, Kennedy-Martin T, Greiner W, et al. Which multi-attribute utility instruments are recommended for use in cost-utility analysis? A review of national health technology assessment (HTA) guidelines. Eur J Health Econ. 2020;21:1245-57.

[11] Quittner AL, Buu A, Messer MA, Modi AC, Watrous M. Development and validation of The Cystic Fibrosis Questionnaire in the United States: a health-related quality-of-life measure for cystic fibrosis. Chest. 2005;128:2347-54.

[12] Quittner AL, Sawicki GS, McMullen A, Rasouliyan L, Pasta DJ, Yegin A, et al. Erratum to: Psychometric evaluation of the Cystic Fibrosis Questionnaire-Revised in a national, US sample. Qual Life Res. 2012;21:1279-90.

[13] Brazier JE, Roberts J. The estimation of a preference-based measure of health from the SF-12. Med Care. 2004;42:851-9.

[14] Goss CH, Edwards TC, Ramsey BW, Aitken ML, Patrick DL. Patient-reported respiratory symptoms in cystic fibrosis. J Cyst Fibros. 2009;8:245-52.

[15] Wainwright CE, Elborn JS, Ramsey BW, Marigowda G, Huang X, Cipolli M, et al. Lumacaftor-ivacaftor in patients with cystic fibrosis homozygous for Phe508del CFTR. N Engl J Med. 2015;373:220-31.

[16] Taylor-Cousar JL, Munck A, McKone EF, van der Ent CK, Moeller A, Simard C, et al. Tezacaftor-ivacaftor in patients with cystic fibrosis homozygous for Phe508del. N Engl J Med. 2017;377:2013-23.

[17] Cohen J. A power primer. Psychol Bull. 1992;112:155-9.

[18] Mokkink LB, Terwee CB, Patrick DL, Alonso J, Stratford PW, Knol DL, et al. The COSMIN study reached international consensus on taxonomy, terminology, and definitions of measurement properties for health-related patient-reported outcomes. J Clin Epidemiol. 2010;63:737-45.

[19] De Vet HCW, Terwee CB, Mokkink LB, Knol DL. Measurement in Medicine: A Practical Guide. Measurement in Medicine: A Practical Guide. Cambridge University Press. 2011: 1-338.

[20] Fayers P, Machin D. Quality of life: the assessment, analysis and interpretation of patient-reported outcomes. 2nd ed. Chichester, UK: Wiley 2007.

[21] Janssen MF, Szende A, Cabases J, Ramos-Goñi JM, Vilagut G, König HH. Population norms for the EQ-5D-3L: a cross-country analysis of population surveys for 20 countries. Eur J Health Econ. 2019;20:205-16.

[22] Rowen D, Brazier J, Ara R, Azzabi Zouraq I. The role of condition-specific preference-based measures in health technology assessment. Pharmacoeconomics. 2017;35:33-41.

**Table 1. Known-group validity based on clinical and demographic variables at baseline and follow-up in the** **EQ-5D-3L trials^a^**

|  |  | **CFQ-R-8D** | | | | **EQ-5D-3L** | | | |
| --- | --- | --- | --- | --- | --- | --- | --- | --- | --- |
| **Variable** | **Groups** | **N** | **Mean** | **SD** | **ES^b^** | **N** | **Mean** | **SD** | **ES^b^** |
| ppFEV_1_—baseline | Normal ≥70% | 246 | 0.842 | 0.11 | 0.31 | 246 | 0.920 | 0.14 | 0.09 |
|  | Mild 40 to <70% | 660 | 0.806 | 0.11 | 0.23 | 660 | 0.908 | 0.12 | 0.06 |
|  | Severe <40% | 80 | 0.78 | 0.13 |  | 80 | 0.900 | 0.12 |  |
|  |  |  | F(2,983) = 12.3 | | |  | F(2,983) = 1.1 | | |
|  |  |  | *P*<0.001 | | |  | *P*=0.34 | | |
| ppFEV_1_—24 weeks^c^ | Normal ≥70% | 277 | 0.863 | 0.11 | 0.48 | 277 | 0.935 | 0.12 | 0.23 |
|  | Mild 40 to <70% | 579 | 0.802 | 0.13 | 0.42 | 579 | 0.903 | 0.15 | 0.35 |
|  | Severe <40% | 63 | 0.749 | 0.14 |  | 63 | 0.854 | 0.13 |  |
|  |  |  | F(2,916) = 33.8 | | |  | F(2,916) = 10.6 | | |
|  |  |  | *P*<0.001 | | |  | *P*<0.001 | | |
| Pulmonary exacerbations—20-24 weeks (≥4)^c^ | No | 835 | 0.825 | 0.12 | 0.60 | 835 | 0.917 | 0.13 | 0.44 |
|  | Yes | 116 | 0.748 | 0.14 |  | 116 | 0.856 | 0.18 |  |
|  |  |  | *t*(949) = 6.3 | | |  | *t*(949) = 4.5 | | |
|  |  |  | *P*<0.001 | | |  | *P*<0.001 | | |
| Age groups—baseline | 14 to <18 years | 191 | 0.837 | 0.11 | 0.26 | 191 | 0.941 | 0.10 | 0.29 |
|  | ≥18 years | 806 | 0.807 | 0.12 |  | 806 | 0.903 | 0.14 |  |
|  |  |  | *t*(995) = 3.3 | | |  | *t*(995) = 3.6 | | |
|  |  |  | *P*=0.001 | | |  | *P*<0.001 | | |

CFQ-R-8D indicates Cystic Fibrosis Questionnaire–Revised–8 Dimensions; ES, effect sizes; ppFEV_1_, percent predicted forced expiratory volume in 1 second.

^a^ Pooled treatment and placebo arms.

^b^ ES was calculated by comparing the utility values for adjacent subgroups in the clinical measures. Small ES ≥0.2 to <0.5; medium ES ≥0.5 to <0.8; large ES ≥0.8.

^c^ 20-24 weeks and 24 weeks indicate follow-up at 20 to 24 weeks and follow-up at 24 weeks, respectively.**Table 2. Known-group validity based on clinical and demographic variables at baseline and follow-up for the SF-6D trial^a^**

|  |  | **CFQ-R-8D** | | | | **SF-6D** | | | | **CFRSD** | | | |
| --- | --- | --- | --- | --- | --- | --- | --- | --- | --- | --- | --- | --- | --- |
| **Variable** | **Groups** | **N** | **Mean** | **SD** | **ES^b^** | **N** | **Mean** | **SD** | **ES^b^** | **N** | **Mean** | **SD** | **ES^b^** |
| ppFEV_1_—baseline | Normal ≥70% | 111 | 0.833 | 0.10 | 0.27 | 111 | 0.822 | 0.12 | 0.18 | 111 | 32.31 | 12.39 | −0.40 |
|  | Mild 40 to <70% | 284 | 0.801 | 0.12 | 0.49 | 284 | 0.801 | 0.12 | 0.34 | 284 | 36.92 | 10.62 | −0.43 |
|  | Severe <40% | 45 | 0.743 | 0.12 |  | 45 | 0.761 | 0.12 |  | 45 | 41.84 | 11.18 |  |
|  |  |  | F(2,437) = 9.6 | | |  | F(2,437) = 4.4 | | |  | F(2,437) = 13.1 | | |
|  |  |  | *P*<0.001 | | |  | *P*=0.013 | | |  | *P*<0.001 | | |
| ppFEV_1_—24 weeks^c^ | Normal ≥70% | 110 | 0.843 | 0.11 | 0.27 | 110 | 0.834 | 0.12 | 0.19 | 110 | 29.68 | 13.70 | −0.48 |
|  | Mild 40 to <70% | 251 | 0.809 | 0.12 | 0.44 | 251 | 0.81 | 0.12 | 0.35 | 251 | 35.82 | 11.44 | −0.64 |
|  | Severe <40% | 45 | 0.754 | 0.15 |  | 45 | 0.766 | 0.13 |  | 45 | 43.96 | 11.67 |  |
|  |  |  | F(2,403) = 8.3 | | |  | F(2,403) = 5.0 | | |  | F(2,403) = 23.5 | | |
|  |  |  | *P*<0.001 | | |  | *P*=0.007 | | |  | *P*<0.001 | | |
| Pulmonary exacerbations—20-24 weeks (≥4)^c^ | No | 368 | 0.822 | 0.12 | 0.70 | 368 | 0.822 | 0.12 | 0.68 | 368 | 34.27 | 12.79 | −0.50 |
|  | Yes | 48 | 0.733 | 0.13 |  | 48 | 0.736 | 0.12 |  | 48 | 40.79 | 12.78 |  |
|  |  |  | *t*(414) = 4.6 | | |  | *t*(414) = 4.5 | | |  | *t*(414) = −3.3 | | |
|  |  |  | *P*<0.001 | | |  | *P*<0.001 | | |  | *P*=0.001 | | |
| Age groups—baseline | 14 to <18 years | 66 | 0.842 | 0.10 | 0.39 | 66 | 0.839 | 0.11 | 0.36 | 66 | 28.14 | 12.42 | −0.84 |
|  | ≥18 years | 375 | 0.796 | 0.12 |  | 375 | 0.796 | 0.12 |  | 375 | 37.69 | 10.65 |  |
|  |  |  | *t*(439) = 2.9 | | |  | *t*(439) = 2.8 | | |  | *t*(439) = −6.7 | | |
|  |  |  | *P*=0.004 | | |  | *P*=0.006 | | |  | *P*<0.001 | | |

CFQ-R-8D indicates Cystic Fibrosis Questionnaire–Revised–8 Dimensions; CFRSD, Cystic Fibrosis Respiratory Symptom Diary; ES, effect sizes; ppFEV_1_, percent predicted forced expiratory volume in 1 second; SF-6D, Short Form 6 dimensions.

^a^ Pooled treatment and placebo arms.

^b^ ES was calculated by comparing the utility values for adjacent sub-groups in the clinical measures. Small ES ≥0.2 to <0.5; medium ES ≥0.5 to <0.8; large ES ≥0.8.

^c^ 20-24 weeks and 24 weeks indicate follow-up at 20 to 24 weeks and follow-up at 24 weeks, respectively.

**Table 3. Responsiveness of generic and condition-specific measures by change in ppFEV_1_ and severity group from baseline to follow up^a-c^**

|  |  | **Change in CFQ-R-8D** | | | | | **Change in EQ-5D-3L or SF-6D** | | | | | **Change in CFRSD** | | | | |
| --- | --- | --- | --- | --- | --- | --- | --- | --- | --- | --- | --- | --- | --- | --- | --- | --- |
| **Variable** | **Groups** | **N** | **Mean** | **SD** | **SRM** | **ES** | **N** | **Mean** | **SD** | **SRM** | **ES** | **N** | **Mean** | **SD** | **SRM** | **ES** |
| **EQ-5D-3L trials** | |  |  |  |  |  |  |  |  |  |  |  |  |  |  |  |
| ppFEV_1_—baseline to 24 weeks^d,e^ | Improvement | 396 | 0.024 | 0.10 | 0.23 | 0.22 | 396 | 0.019 | 0.14 | 0.14 | 0.25 | **–** | **–** | **–** | **–** | **–** |
|  | No change | 243 | 0.001 | 0.09 | 0.01 | 0.23 | 243 | −0.016 | 0.13 | −0.12 | 0.06 | **–** | **–** | **–** | **–** | **–** |
|  | Deterioration | 267 | −0.023 | 0.11 | −0.21 |  | 267 | −0.024 | 0.15 | −0.16 |  | **–** | **–** | **–** | **–** |  |
| ppFEV_1_ severity group—baseline  to 24 weeks^d^ | Improvement | 120 | 0.041 | 0.10 | 0.41 | 0.39 | 120 | 0.035 | 0.13 | 0.28 | 0.30 | **–** | **–** | **–** | **–** | **–** |
|  | No change | 723 | 0.000 | 0.11 | 0.00 | 0.28 | 723 | −0.008 | 0.14 | −0.06 | 0.05 | **–** | **–** | **–** | **–** | **–** |
|  | Deterioration | 63 | −0.029 | 0.10 | −0.28 |  | 63 | −0.015 | 0.16 | −0.09 |  | **–** | **–** | **–** | **–** |  |
| Pulmonary exacerbations (≥4) | No | 829 | 0.011 | 0.10 | 0.11 | 0.59 | 829 | 0.006 | 0.13 | 0.05 | 0.37 | **–** | **–** | **–** | **–** | **–** |
|  | Yes | 116 | −0.051 | 0.14 | −0.49 |  | 116 | −0.046 | 0.20 | −0.32 |  | **–** | **–** | **–** | **–** |  |
| **SF-6D trial** |  |  |  |  |  |  |  |  |  |  |  |  |  |  |  |  |
| ppFEV_1_—baseline to 24 weeks^d,e^ | Improvement | 155 | 0.030 | 0.09 | 0.33 | 0.40 | 155 | 0.026 | 0.11 | 0.23 | 0.23 | 155 | 3.79 | 10.50 | 0.36 | 0.38 |
|  | No change | 112 | −0.010 | 0.11 | −0.09 | −0.06 | 112 | 0.002 | 0.10 | 0.02 | 0.09 | 112 | −0.28 | 9.58 | −0.03 | 0.04 |
|  | Deterioration | 128 | −0.004 | 0.10 | −0.04 |  | 128 | −0.007 | 0.09 | −0.08 |  | 128 | −0.74 | 11.34 | −0.07 |  |
| ppFEV_1_ severity group—baseline  to 24 weeks^d^ | Improvement | 39 | 0.027 | 0.09 | 0.31 | 0.19 | 39 | 0.056 | 0.13 | 0.42 | 0.48 | 39 | 4.87 | 9.36 | 0.52 | 0.36 |
|  | No change | 320 | 0.008 | 0.10 | 0.08 | 0.24 | 320 | 0.006 | 0.10 | 0.06 | 0.26 | 320 | 0.98 | 10.73 | 0.09 | 0.20 |
|  | Deterioration | 36 | −0.016 | 0.11 | −0.15 |  | 36 | −0.021 | 0.08 | −0.28 |  | 36 | −1.14 | 11.29 | −0.10 |  |
| Pulmonary exacerbations (≥4) | No | 357 | 0.012 | 0.10 | 0.12 | 0.42 | 357 | 0.014 | 0.11 | 0.13 | 0.50 | 357 | 1.57 | 10.67 | 0.15 | 0.24 |
|  | Yes | 48 | −0.031 | 0.12 | −0.30 |  | 48 | −0.039 | 0.10 | −0.37 |  | 48 | −1.02 | 11.67 | −0.09 |  |

CFQ-R-8D indicates Cystic Fibrosis Questionnaire–Revised–8 Dimensions; CFRSD, Cystic Fibrosis Respiratory Symptom Diary; ES, effect sizes; ppFEV_1_, percent predicted forced expiratory volume in 1 second; SF-6D, Short Form 6 dimensions; SRM, standardized response mean.

^a^ Pooled treatment and placebo arms.

^b^ Change in ppFEV_1_ severity group was defined as movement between the following groups: ppFEV_1_ <40%, ≥40 to <70%, and ≥70%.

^c^ Small SRM/ES ≥0.2 to <0.5; medium SRM/ES ≥0.5 to <0.8; large SRM/ES ≥0.8.

^d^ 24 weeks indicates follow-up at 24 weeks.

^e^ For ppFEV_1_, an improvement was defined as an increase of ≥2 percentage points, no change was defined as a change of <±2 percentage points, and deterioration was defined as a decrease of ≥2 percentage points.

**Table 4. Floor and ceiling of generic and condition-specific measures at baseline and week 24^a^**

|  | **EQ-5D-3L trials** | | | | **SF-6D trial** | | | |
| --- | --- | --- | --- | --- | --- | --- | --- | --- |
|  | % at floor | | % at ceiling | | % at floor | | % at ceiling | |
|  | Baseline | 24 weeks^b^ | Baseline | 24 weeks^b^ | Baseline | 24 weeks^b^ | Baseline | 24 weeks^b^ |
| **N** | 997 | 951 | 997 | 951 | 441 | 441 | 416 | 416 |
| **CFQ-R-8D** |  |  |  |  |  |  |  |  |
| CFQ-R-8D utility | 0.00 | 0.00 | 1.70 | 3.68 | 0.00 | 0.00 | 1.81 | 3.85 |
| Physical Functioning | 0.70 | 1.89 | 72.42 | 69.09 | 1.36 | 2.16 | 63.04 | 64.18 |
| Role Functioning | 0.90 | 2.52 | 73.62 | 69.93 | 1.81 | 1.20 | 70.29 | 74.52 |
| Emotion | 1.00 | 0.63 | 39.92 | 50.37 | 0.91 | 1.20 | 40.36 | 45.67 |
| Vitality | 1.50 | 1.47 | 37.61 | 40.48 | 1.81 | 1.92 | 41.72 | 42.55 |
| Breathing Difficulty | 0.70 | 0.32 | 53.86 | 52.68 | 0.23 | 0.72 | 51.93 | 57.69 |
| Cough | 10.53 | 9.67 | 5.22 | 10.09 | 9.30 | 8.17 | 4.31 | 6.73 |
| Abdominal Pain | 0.60 | 0.74 | 59.58 | 58.99 | 0.68 | 1.20 | 53.74 | 53.37 |
| Body Image | 1.20 | 1.26 | 64.19 | 64.25 | 1.36 | 1.92 | 64.63 | 62.74 |
| **EQ-5D-3L** |  |  |  |  |  |  |  |  |
| EQ-5D-3L utility | 0.00 | 0.00 | 60.88 | 62.36 | – | – | – | – |
| Mobility | 0.00 | 0.00 | 95.39 | 93.06 | – | – | – | – |
| Self-care | 0.00 | 0.11 | 99.70 | 99.37 | – | – | – | – |
| Usual activities | 0.10 | 0.21 | 88.37 | 85.59 | – | – | – | – |
| Pain/ discomfort | 0.40 | 0.74 | 73.62 | 75.81 | – | – | – | – |
| Depression/ anxiety | 0.40 | 0.42 | 80.74 | 81.49 | – | – | – | – |
| **SF-6D** |  |  |  |  |  |  |  |  |
| SF-6D utility | – | – | – | – | 0.00 | 0.00 | 5.44 | 8.65 |
| Physical functioning | – | – | – | – | 8.16 | 7.71 | 55.56 | 61.20 |
| Role limitation | – | – | – | – | 23.81 | 22.84 | 48.53 | 52.64 |
| Social functioning | – | – | – | – | 0.68 | 0.24 | 63.04 | 64.90 |
| Pain | – | – | – | – | 0.45 | 0.72 | 74.15 | 73.08 |
| Mental health | – | – | – | – | 0.68 | 0.24 | 55.33 | 58.17 |
| Energy | – | – | – | – | 5.44 | 5.53 | 7.48 | 10.10 |
| **CFRSD** | – | – | – | – | 0.00 | 0.00 | 1.81 | 3.85 |

CFQ-R-8D indicates Cystic Fibrosis Questionnaire–Revised–8 Dimensions; CFRSD, Cystic Fibrosis Respiratory Symptom Diary; SF-6D, Short Form 6 dimensions.

^a^ Pooled treatment and placebo arms.

^b^ 24 weeks indicates follow-up at 24 weeks.

**Table 5. Frequencies of CFQ-R-8D when generic measures equal 1 (ie, no problems in any dimension) at baseline**

|  | **Level 1** | **Level 2** | **Level 3** | **Level 4** |
| --- | --- | --- | --- | --- |
| **EQ-5D-3L trials (EQ-5D-3L = 1) n = 607/997** | | | | |
| **CFQ-R-8D, n** |  |  |  |  |
| Physical Functioning | 515 | 81 | 11 | 0 |
| Vitality | 281 | 303 | 20 | 3 |
| Emotion | 314 | 277 | 12 | 4 |
| Role Functioning | 507 | 87 | 12 | 1 |
| Cough | 42 | 302 | 227 | 36 |
| Breathing Difficulty | 408 | 193 | 6 | 0 |
| Abdominal Pain | 422 | 178 | 7 | 0 |
| Body Image^a^ | 459 | 112 | 34 | 2 |
| **SF-6D trial (SF-6D = 1) n = 24/413** | | | | |
| **CFQ-R-8D, n** |  |  |  |  |
| Physical Functioning | 21 | 3 | 0 | 0 |
| Vitality | 18 | 6 | 0 | 0 |
| Emotion | 21 | 3 | 0 | 0 |
| Role Functioning | 21 | 2 | 0 | 1 |
| Cough | 4 | 15 | 4 | 1 |
| Breathing Difficulty | 20 | 4 | 0 | 0 |
| Abdominal Pain | 16 | 7 | 1 | 0 |
| Body Image^a^ | 22 | 1 | 0 | 1 |

CFQ-R-8D, Cystic Fibrosis Questionnaire–Revised–8 Dimensions; SF-6D, Short Form 6 dimensions.

^a^ Body Image has only two levels in the classifier but is based on an item with four levels (ie, very true, somewhat true, somewhat false, and very false). Disutility is associated with “somewhat true = 3” and “very false = 4.”

**Fig. 1 Distribution of utility measures at baseline and week 24^a^**


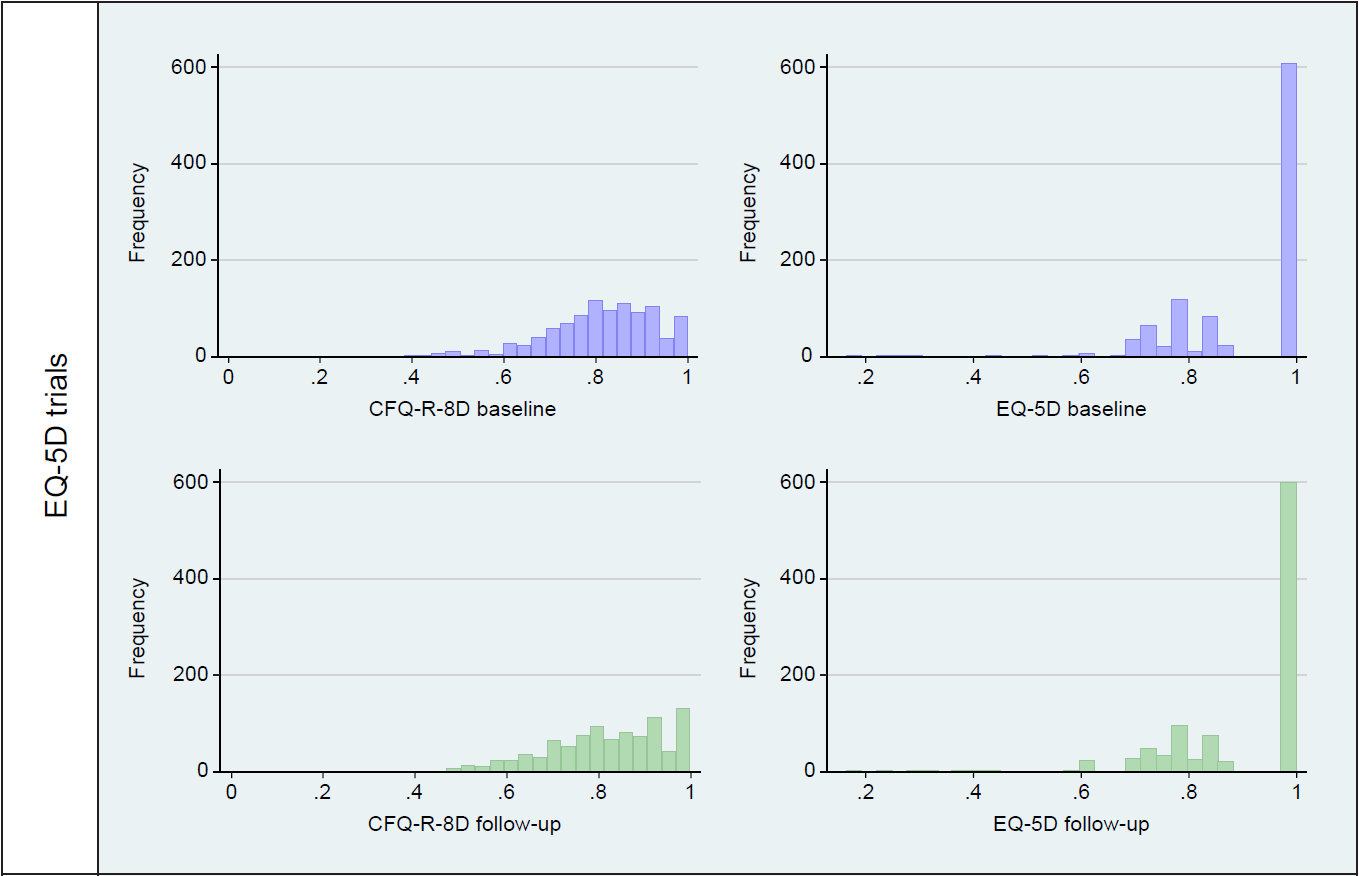


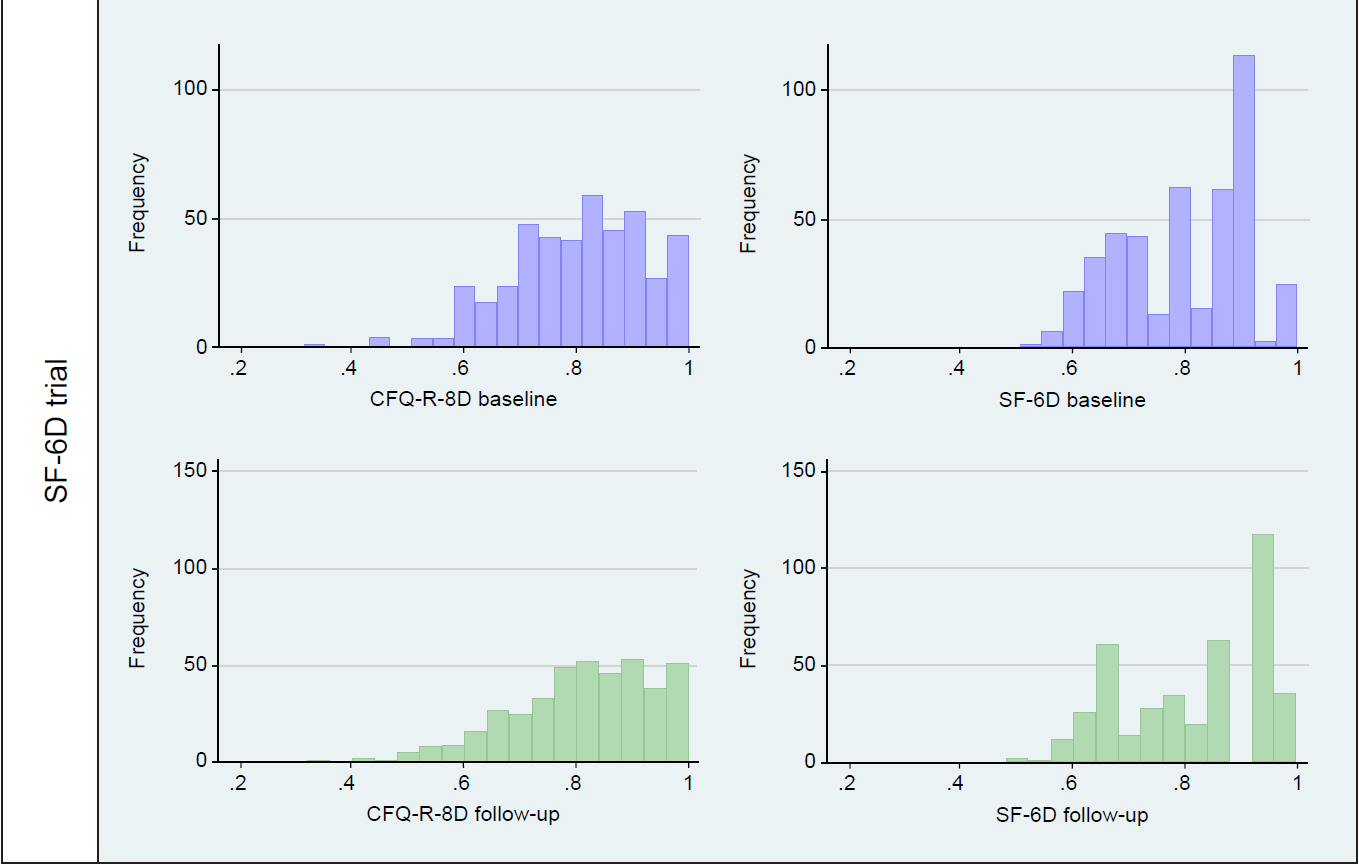


CFQ-R-8D, Cystic Fibrosis Questionnaire–Revised–8 Dimensions; SF-6D, Short Form 6 dimensions.

^a^ Pooled treatment and placebo arms.
