# Supplementary material for: Psychometric performance of the CFQ-R-8D compared to the EQ-5D-3L and SF-6D in people with cystic fibrosis
Source: J Patient Rep Outcomes. 2024 Feb 28;8:24. doi: 10.1186/s41687-024-00697-w (PMC10900011; doi:10.1186/s41687-024-00697-w)
Supplement: Supplementary file 3 — Supplementary Material 3 [file 41687_2024_697_MOESM3_ESM.docx]

**Psychometric Performance of the CFQ-R-8D Compared to the EQ-5D-3L and SF-6D in People With Cystic Fibrosis**

**Table of Contents**

[Supplementary Table 1. Baseline and follow-up measures (for each trial data set, comprising pooled treatment and placebo arms) 2](#_Toc153436327)

[Supplementary Table 2. Pearson and Spearman rank correlation at baseline for EQ-5D trials](#_Toc153436328)^[a](#_Toc153436328)^ [3](#_Toc153436328)

[Supplementary Table 3. Pearson and Spearman rank correlations at follow-up for EQ-5D trials](#_Toc153436329)^[a](#_Toc153436329)^ [4](#_Toc153436329)

[Supplementary Table 4. Pearson and Spearman rank correlation at baseline for SF-6D trial](#_Toc153436330)^[a](#_Toc153436330)^ [5](#_Toc153436330)

[Supplementary Table 5. Pearson and Spearman rank correlation at follow-up for SF-6D trials](#_Toc153436331)^[a](#_Toc153436331)^ [6](#_Toc153436331)

[Supplementary Table 6. Pearson and Spearman rank correlation of change for EQ-5D trials](#_Toc153436332)^[a](#_Toc153436332)^ [8](#_Toc153436332)

[Supplementary Table 7. Pearson and Spearman rank correlation of change for SF-6D trials](#_Toc153436333)^[a](#_Toc153436333)^ [9](#_Toc153436333)

[Supplementary Table 8. Responsiveness of generic and condition-specific measure from baseline to follow-up 11](#_Toc153436334)

# Supplementary Table 1. Baseline and follow-up measures (for each trial data set, comprising pooled treatment and placebo arms)

|  |  | **EQ-5D trials**  **baseline: N = 997;  24 weeks^a^: N = 951^b^** | | **SF-6D trial**  **baseline N = 441;  24 weeks^a^: N = 416** | |
| --- | --- | --- | --- | --- | --- |
|  |  | **Mean** | **SD** | **Mean** | **SD** |
| **CFQ-R-8D** | Baseline | 0.812 | 0.12 | 0.803 | 0.12 |
|  | 24 weeks | 0.816 | 0.13 | 0.811 | 0.13 |
| **EQ-5D-3L** | Baseline | 0.910 | 0.13 | **–** | **–** |
|  | 24 weeks | 0.909 | 0.14 | **–** | **–** |
| **SF-6D** | Baseline | **–** | **–** | 0.802 | 0.12 |
|  | 24 weeks | **–** | **–** | 0.812 | 0.13 |
| **CFQ-R^c^** |  |  |  |  |  |
| Physical Functioning | Baseline | 79.45 | 20.18 | 77.19 | 21.58 |
|  | 24 weeks | 78.03 | 23.08 | 77.76 | 22.31 |
| Vitality | Baseline | 64.56 | 17.44 | 63.38 | 18.40 |
|  | 24 weeks | 63.00 | 19.58 | 62.96 | 20.22 |
| Emotional Functioning | Baseline | 82.78 | 15.25 | 82.38 | 16.35 |
|  | 24 weeks | 83.24 | 16.15 | 82.29 | 16.60 |
| Role Functioning | Baseline | 84.45 | 16.25 | 83.87 | 17.00 |
|  | 24 weeks | 83.80 | 18.04 | 85.18 | 17.05 |
| Respiratory Symptoms | Baseline | 68.23 | 16.96 | 69.27 | 16.22 |
|  | 24 weeks | 69.76 | 18.55 | 70.84 | 17.39 |
| Social Functioning | Baseline | 73.89 | 16.86 | 73.14 | 16.65 |
|  | 24 weeks | 71.58 | 18.23 | 73.04 | 16.92 |
| Health Perceptions | Baseline | 66.98 | 20.54 | 64.53 | 20.78 |
|  | 24 weeks | 66.21 | 21.24 | 64.85 | 21.27 |
| Body Image | Baseline | 77.58 | 22.15 | 76.77 | 22.38 |
|  | 24 weeks | 79.10 | 22.29 | 77.70 | 22.85 |
| Digestion | Baseline | 83.47 | 16.82 | 82.19 | 16.58 |
|  | 24 weeks | 82.46 | 17.61 | 81.76 | 17.23 |
| Weight | Baseline | 74.47 | 33.84 | 75.06 | 31.74 |
|  | 24 weeks | 76.38 | 31.54 | 75.96 | 31.54 |
| Eating Disturbance | Baseline | 92.37 | 15.08 | 90.73 | 17.38 |
|  | 24 weeks | 90.73 | 16.64 | 89.88 | 18.14 |
| Treatment Burden | Baseline | 56.64 | 19.27 | 60.24 | 19.44 |
|  | 24 weeks | 58.47 | 18.97 | 60.95 | 19.19 |
| **CFRSD^c^** | Baseline | – | – | 36.26 | 11.44 |
|  | 24 weeks | – | – | 35.02 | 12.94 |

CFQ-R-8D, Cystic Fibrosis Questionnaire–Revised–8 Dimensions; CFRSD, Cystic Fibrosis Respiratory Symptom Diary; SF-6D, Short Form 6 dimensions.

^a^ 24 weeks = follow-up at 24 weeks.

^b^ Sample size for CFQ-R scores vary by dimension as scores can be calculated even when some questions have not been completed.

^c^ CFQ-R and CFRSD scores for those with both CFQ-R-8D and EQ-5D-3L or SF-6D scores.

# Supplementary Table 2. Pearson and Spearman rank correlation at baseline for EQ-5D trials^a^

|  | **Physical Functioning** | **Vitality** | **Emotional Functioning** | **Role Functioning** | **Respiratory Symptoms** | **Digestion** | **Body Image** | **Social Functioning** | **Health Perceptions** | **Weight** | **Eat** | **Treatment Burden** |
| --- | --- | --- | --- | --- | --- | --- | --- | --- | --- | --- | --- | --- |
| CFQ-R-8D dimensions |  |  |  |  |  |  |  |  |  |  |  |  |
| Physical Functioning | **-0.67** | -0.45 | -0.35 | -0.38 | -0.36 | -0.23 | -0.19 | -0.32 | -0.43 | -0.07 | -0.19 | -0.24 |
| Vitality | -0.46 | **-0.72** | -0.32 | -0.37 | -0.37 | -0.27 | -0.24 | -0.32 | -0.35 | -0.10 | -0.19 | -0.22 |
| Emotional Functioning | -0.32 | -0.39 | **-0.71** | -0.27 | -0.27 | -0.22 | -0.24 | -0.31 | -0.31 | -0.08 | -0.10 | -0.28 |
| Role Functioning | -0.28 | -0.28 | -0.31 | **-0.62** | -0.23 | -0.16 | -0.16 | -0.24 | -0.29 | -0.11 | -0.16 | -0.23 |
| Cough | -0.37 | -0.36 | -0.24 | -0.23 | **-0.80** | -0.20 | -0.19 | -0.30 | -0.36 | -0.17 | -0.11 | -0.22 |
| Breathing Difficulty | -0.55 | -0.48 | -0.33 | -0.37 | **-0.64** | -0.24 | -0.23 | -0.33 | -0.46 | -0.15 | -0.15 | -0.28 |
| Abdominal Pain | -0.21 | -0.26 | -0.21 | -0.19 | -0.18 | **-0.73** | -0.12 | -0.18 | -0.20 | -0.06 | -0.13 | -0.12 |
| Body Image | -0.34 | -0.34 | -0.43 | -0.31 | -0.24 | -0.18 | **-0.68** | -0.34 | -0.40 | -0.15 | -0.21 | -0.23 |
| CFQ-R-8D utility | 0.70 | 0.73 | 0.64 | 0.64 | 0.69 | 0.50 | 0.37 | 0.51 | 0.59 | 0.22 | 0.24 | 0.39 |
|  |  |  |  |  |  |  |  |  |  |  |  |  |
| EQ-5D-3L dimensions |  |  |  |  |  |  |  |  |  |  |  |  |
| Mobility | -0.29 | -0.24 | -0.15 | -0.25 | -0.19 | -0.09 | -0.15 | -0.19 | -0.23 | -0.03 | -0.12 | -0.08 |
| Self-care | -0.09 | -0.03 | -0.04 | -0.07 | -0.02 | -0.03 | -0.03 | -0.04 | 0.00 | -0.03 | -0.06 | 0.02 |
| Usual activities | -0.39 | -0.34 | -0.35 | -0.45 | -0.29 | -0.21 | -0.21 | -0.30 | -0.37 | -0.08 | -0.17 | -0.23 |
| Pain/ discomfort | -0.37 | -0.35 | -0.28 | -0.32 | -0.27 | -0.28 | -0.15 | -0.27 | -0.34 | -0.01 | -0.16 | -0.20 |
| Anxiety/ depression | -0.28 | -0.29 | -0.47 | -0.26 | -0.19 | -0.22 | -0.23 | -0.29 | -0.30 | -0.08 | -0.14 | -0.16 |
| EQ-5D-3L utility | 0.50 | 0.47 | 0.49 | 0.46 | 0.37 | 0.36 | 0.25 | 0.40 | 0.47 | 0.06 | 0.20 | 0.26 |

CFQ-R-8D, Cystic Fibrosis Questionnaire–Revised–8 Dimensions.

^a^ Pearson correlations were used for continuous data; Spearman correlations were used for ordinal data (domains); weak <0.3; moderate <0.5 to ≥0.3; strong ≥0.5.

# Supplementary Table 3. Pearson and Spearman rank correlations at follow-up for EQ-5D trials^a^

|  | **Physical Functioning** | **Vitality** | **Emotional Functioning** | **Role Functioning** | **Respiratory Symptoms** | **Digestion** | **Body Image** | **Social Functioning** | **Health Perceptions** | **Weight** | **Eat** | **Treatment Burden** |
| --- | --- | --- | --- | --- | --- | --- | --- | --- | --- | --- | --- | --- |
| CFQ-R-8D dimensions |  |  |  |  |  |  |  |  |  |  |  |  |
| Physical Functioning | **-0.73** | -0.47 | -0.36 | -0.44 | -0.43 | -0.22 | -0.25 | -0.37 | -0.45 | -0.09 | -0.30 | -0.28 |
| Vitality | -0.54 | **-0.78** | -0.43 | -0.47 | -0.44 | -0.33 | -0.30 | -0.43 | -0.45 | -0.13 | -0.29 | -0.28 |
| Emotional Functioning | -0.33 | -0.41 | **-0.76** | -0.34 | -0.33 | -0.32 | -0.33 | -0.38 | -0.38 | -0.10 | -0.20 | -0.28 |
| Role Functioning | -0.42 | -0.39 | -0.33 | **-0.72** | -0.34 | -0.18 | -0.19 | -0.35 | -0.39 | -0.08 | -0.27 | -0.24 |
| Cough | -0.44 | -0.42 | -0.28 | -0.32 | **-0.82** | -0.15 | -0.21 | -0.32 | -0.42 | -0.22 | -0.24 | -0.24 |
| Breathing Difficulty | -0.64 | -0.53 | -0.39 | -0.43 | **-0.68** | -0.22 | -0.32 | -0.42 | -0.52 | -0.14 | -0.29 | -0.33 |
| Abdominal Pain | -0.24 | -0.30 | -0.25 | -0.22 | -0.18 | **-0.75** | -0.18 | -0.24 | -0.24 | -0.02 | -0.20 | -0.15 |
| Body Image | -0.41 | -0.39 | -0.48 | -0.30 | -0.29 | -0.28 | **-0.76** | -0.47 | -0.42 | -0.17 | -0.29 | -0.29 |
| CFQ-R-8D utility | 0.77 | 0.75 | 0.65 | 0.69 | 0.71 | 0.49 | 0.42 | 0.57 | 0.65 | 0.19 | 0.41 | 0.42 |
|  |  |  |  |  |  |  |  |  |  |  |  |  |
| EQ-5D-3L dimensions |  |  |  |  |  |  |  |  |  |  |  |  |
| Mobility | -0.40 | -0.34 | -0.22 | -0.34 | -0.29 | -0.11 | -0.12 | -0.22 | -0.30 | -0.04 | -0.20 | -0.18 |
| Self-care | -0.08 | -0.03 | -0.02 | -0.06 | -0.03 | -0.02 | -0.03 | -0.06 | -0.07 | 0.02 | -0.07 | -0.05 |
| Usual activities | -0.49 | -0.44 | -0.34 | -0.50 | -0.35 | -0.21 | -0.18 | -0.34 | -0.42 | -0.08 | -0.28 | -0.29 |
| Pain/ discomfort | -0.39 | -0.39 | -0.29 | -0.33 | -0.32 | -0.32 | -0.23 | -0.29 | -0.37 | -0.02 | -0.23 | -0.21 |
| Anxiety/ depression | -0.28 | -0.37 | -0.50 | -0.31 | -0.24 | -0.28 | -0.31 | -0.32 | -0.31 | -0.13 | -0.21 | -0.21 |
| EQ-5D-3L utility | 0.55 | 0.52 | 0.46 | 0.48 | 0.44 | 0.37 | 0.28 | 0.39 | 0.48 | 0.13 | 0.32 | 0.31 |

CFQ-R-8D, Cystic Fibrosis Questionnaire–Revised–8 Dimensions.

^a^ Pearson correlations were used for continuous data; Spearman correlations were used for ordinal data (domains); weak <0.3; moderate <0.5 to ≥0.3; strong ≥0.5.

# Supplementary Table 4. Pearson and Spearman rank correlation at baseline for SF-6D trial^a^

|  | **Physical Functioning** | **Vitality** | **Emotional Functioning** | **Role Functioning** | **Respiratory Symptoms** | **Digestion** | **Body Image** | **Social Functioning** | **Health Perceptions** | **Weight** | **Eat** | **Treatment Burden** | **CFRSD** |
| --- | --- | --- | --- | --- | --- | --- | --- | --- | --- | --- | --- | --- | --- |
| CFQ-R-8D dimensions |  |  |  |  |  |  |  |  |  |  |  |  |  |
| Physical Functioning | **-0.77** | -0.38 | -0.36 | -0.42 | -0.48 | -0.21 | -0.20 | -0.37 | -0.44 | -0.16 | -0.23 | -0.29 | 0.43 |
| Vitality | -0.50 | **-0.75** | -0.46 | -0.49 | -0.45 | -0.22 | -0.24 | -0.40 | -0.42 | -0.10 | -0.29 | -0.28 | 0.52 |
| Emotional Functioning | -0.29 | -0.39 | **-0.71** | -0.30 | -0.27 | -0.29 | -0.19 | -0.29 | -0.34 | -0.10 | -0.21 | -0.20 | 0.29 |
| Role Functioning | -0.30 | -0.32 | -0.28 | **-0.65** | -0.23 | -0.24 | -0.13 | -0.34 | -0.31 | -0.14 | -0.21 | -0.22 | 0.27 |
| Cough | -0.45 | -0.38 | -0.23 | -0.30 | **-0.77** | -0.28 | -0.24 | -0.28 | -0.33 | -0.20 | -0.13 | -0.21 | 0.62 |
| Breathing Difficulty | -0.53 | -0.42 | -0.36 | -0.37 | **-0.64** | -0.24 | -0.23 | -0.34 | -0.41 | -0.15 | -0.18 | -0.31 | 0.57 |
| Abdominal Pain | -0.23 | -0.27 | -0.24 | -0.31 | -0.28 | **-0.76** | -0.14 | -0.20 | -0.22 | -0.07 | -0.18 | -0.23 | 0.29 |
| Body Image | -0.31 | -0.27 | -0.41 | -0.31 | -0.27 | -0.21 | **-0.67** | -0.37 | -0.33 | -0.18 | -0.24 | -0.29 | 0.27 |
| CFQ-R-8D utility | 0.69 | 0.68 | 0.64 | 0.68 | 0.71 | 0.52 | 0.36 | 0.53 | 0.58 | 0.21 | 0.33 | 0.41 | -0.67 |
|  |  |  |  |  |  |  |  |  |  |  |  |  |  |
| SF-6D dimensions |  |  |  |  |  |  |  |  |  |  |  |  |  |
| Physical functioning | **-0.70** | -0.42 | -0.32 | -0.45 | -0.42 | -0.26 | -0.24 | -0.36 | -0.46 | -0.11 | -0.18 | -0.35 | 0.46 |
| Role limitation | -0.50 | -0.47 | -0.54 | **-0.59** | -0.41 | -0.34 | -0.25 | -0.40 | -0.52 | -0.19 | -0.30 | -0.36 | 0.42 |
| Social functioning | -0.40 | -0.46 | -0.51 | -0.47 | -0.33 | -0.28 | -0.23 | -0.38 | -0.49 | -0.26 | -0.32 | -0.33 | 0.37 |
| Pain | -0.30 | -0.34 | -0.26 | -0.33 | -0.32 | -0.36 | -0.22 | -0.25 | -0.27 | -0.20 | -0.23 | -0.19 | 0.33 |
| Mental health | -0.28 | -0.35 | **-0.65** | -0.35 | -0.25 | -0.27 | -0.25 | -0.32 | -0.32 | -0.13 | -0.28 | -0.25 | 0.27 |
| Energy/ vitality | -0.54 | **-0.72** | -0.45 | -0.50 | -0.41 | -0.23 | -0.28 | -0.49 | -0.55 | -0.21 | -0.30 | -0.37 | 0.52 |
| SF-6D utility | 0.53 | 0.59 | 0.67 | 0.61 | 0.43 | 0.34 | 0.34 | 0.47 | 0.59 | 0.25 | 0.34 | 0.41 | -0.50 |

CFQ-R-8D, Cystic Fibrosis Questionnaire–Revised–8 Dimensions; CFRSD Cystic Fibrosis Respiratory Symptom Diary SF-6D, Short Form 6 dimensions.

^a^ Pearson correlations were used for continuous data; Spearman correlations were used for ordinal data (domains); weak <0.3; moderate <0.5 to ≥0.3; strong ≥0.5.

# Supplementary Table 5. Pearson and Spearman rank correlation at follow-up for SF-6D trials^a^

|  | **Physical Functioning** | **Vitality** | **Emotional Functioning** | **Role Functioning** | **Respiratory Symptoms** | **Digestion** | **Body Image** | **Social Functioning** | **Health Perceptions** | **Weight** | **Eat** | **Treatment Burden** | **CFRSD** |
| --- | --- | --- | --- | --- | --- | --- | --- | --- | --- | --- | --- | --- | --- |
| CFQ-R-8D dimensions |  |  |  |  |  |  |  |  |  |  |  |  |  |
| Physical Functioning | **-0.78** | -0.46 | -0.36 | -0.49 | -0.46 | -0.24 | -0.25 | -0.43 | -0.50 | -0.13 | -0.28 | -0.37 | 0.51 |
| Vitality | -0.49 | **-0.76** | -0.42 | -0.43 | -0.48 | -0.31 | -0.21 | -0.39 | -0.45 | -0.09 | -0.24 | -0.33 | 0.58 |
| Emotional Functioning | -0.33 | -0.43 | **-0.77** | -0.41 | -0.35 | -0.33 | -0.19 | -0.31 | -0.38 | -0.14 | -0.27 | -0.28 | 0.37 |
| Role Functioning | -0.32 | -0.32 | -0.30 | **-0.67** | -0.24 | -0.16 | -0.03 | -0.26 | -0.37 | -0.12 | -0.22 | -0.26 | 0.27 |
| Cough | -0.42 | -0.43 | -0.23 | -0.33 | **-0.78** | -0.21 | -0.18 | -0.34 | -0.37 | -0.20 | -0.23 | -0.24 | 0.67 |
| Breathing Difficulty | -0.59 | -0.48 | -0.39 | -0.43 | **-0.65** | -0.27 | -0.25 | -0.36 | -0.50 | -0.12 | -0.24 | -0.23 | 0.59 |
| Abdominal Pain | -0.26 | -0.32 | -0.31 | -0.23 | -0.27 | **-0.76** | -0.15 | -0.25 | -0.31 | -0.10 | -0.20 | -0.25 | 0.30 |
| Body Image | -0.33 | -0.26 | -0.37 | -0.28 | -0.22 | -0.24 | **-0.75** | -0.41 | -0.31 | -0.12 | -0.29 | -0.25 | 0.25 |
| CFQ-R-8D utility | 0.74 | 0.73 | 0.67 | 0.70 | 0.72 | 0.52 | 0.32 | 0.55 | 0.66 | 0.19 | 0.41 | 0.46 | -0.70 |
|  |  |  |  |  |  |  |  |  |  |  |  |  |  |
| SF-6D dimensions |  |  |  |  |  |  |  |  |  |  |  |  |  |
| Physical functioning | **-0.75** | -0.44 | -0.34 | -0.45 | -0.48 | -0.24 | -0.23 | -0.44 | -0.46 | -0.07 | -0.25 | -0.32 | 0.51 |
| Role limitation | -0.54 | -0.54 | -0.62 | **-0.60** | -0.42 | -0.34 | -0.20 | -0.46 | -0.54 | -0.16 | -0.33 | -0.40 | 0.46 |
| Social functioning | -0.46 | -0.54 | -0.59 | -0.49 | -0.39 | -0.29 | -0.23 | -0.49 | -0.51 | -0.19 | -0.39 | -0.34 | 0.47 |
| Pain | -0.36 | -0.32 | -0.29 | -0.34 | -0.20 | -0.30 | -0.17 | -0.27 | -0.31 | -0.10 | -0.23 | -0.19 | 0.28 |
| Mental health | -0.39 | -0.49 | **-0.72** | -0.44 | -0.33 | -0.31 | -0.26 | -0.40 | -0.44 | -0.12 | -0.27 | -0.26 | 0.39 |
| Energy/ vitality | -0.57 | **-0.76** | -0.46 | -0.48 | -0.47 | -0.27 | -0.30 | -0.57 | -0.62 | -0.19 | -0.33 | -0.37 | 0.58 |
| SF-6D utility | 0.59 | 0.70 | 0.70 | 0.62 | 0.49 | 0.39 | 0.31 | 0.58 | 0.64 | 0.18 | 0.39 | 0.42 | -0.55 |

CFQ-R-8D, Cystic Fibrosis Questionnaire–Revised–8 Dimensions; CFRSD, Cystic Fibrosis Respiratory Symptom Diary; SF-6D, Short Form 6 dimensions.

^a^ Pearson correlations were used for continuous data; Spearman correlations were used for ordinal data (domains); weak <0.3; moderate <0.5 to ≥0.3; strong ≥0.5. Bold items – correlations as expected. Italicized – correlations not as expected

# Supplementary Table 6. Pearson and Spearman rank correlation of change for EQ-5D trials^a^

| **Change** | **Physical Functioning** | **Vitality** | **Emotional Functioning** | **Role Functioning** | **Respiratory Symptoms** | **Digestion** | **Body Image** | **Social Functioning** | **Health Perceptions** | **Weight** | **Eat** | **Treatment Burden** |
| --- | --- | --- | --- | --- | --- | --- | --- | --- | --- | --- | --- | --- |
| CFQ-R-8D dimensions |  |  |  |  |  |  |  |  |  |  |  |  |
| Physical Functioning | **0.66** | 0.28 | 0.18 | 0.29 | 0.28 | 0.14 | 0.09 | 0.19 | 0.25 | 0.14 | 0.23 | 0.14 |
| Vitality | 0.35 | **0.63** | 0.17 | 0.25 | 0.27 | 0.15 | 0.13 | 0.16 | 0.27 | 0.10 | 0.20 | 0.11 |
| Emotional Functioning | 0.21 | 0.21 | **0.66** | 0.19 | 0.15 | 0.02 | 0.16 | 0.15 | 0.16 | 0.07 | 0.12 | 0.13 |
| Role Functioning | 0.25 | 0.21 | 0.11 | **0.65** | 0.22 | 0.08 | 0.07 | 0.15 | 0.23 | 0.11 | 0.22 | 0.12 |
| Cough | 0.36 | 0.31 | 0.19 | 0.23 | **0.55** | 0.06 | 0.08 | 0.15 | 0.30 | 0.15 | 0.13 | 0.09 |
| Breathing Difficulty | 0.28 | 0.31 | 0.13 | 0.23 | **0.77** | 0.03 | 0.04 | 0.12 | 0.30 | 0.10 | 0.12 | 0.05 |
| Abdominal Pain | 0.10 | 0.12 | 0.05 | 0.08 | 0.05 | **0.65** | 0.04 | 0.09 | 0.05 | 0.06 | 0.14 | 0.07 |
| Body Image | 0.10 | 0.11 | 0.19 | 0.14 | 0.02 | 0.05 | **0.60** | 0.14 | 0.08 | 0.08 | 0.14 | 0.12 |
| CFQ-R-8D utility | **0.63** | **0.58** | 0.45 | **0.58** | **0.61** | 0.30 | 0.20 | 0.46 | 0.27 | 0.23 | 0.34 | 0.21 |
|  |  |  |  |  |  |  |  |  |  |  |  |  |
| EQ-5D-3L dimensions |  |  |  |  |  |  |  |  |  |  |  |  |
| Mobility | 0.30 | 0.21 | 0.13 | 0.24 | 0.20 | -0.02 | 0.09 | 0.12 | 0.17 | 0.07 | 0.14 | 0.10 |
| Self-care | 0.10 | 0.01 | -0.01 | 0.00 | -0.01 | -0.01 | 0.06 | 0.01 | -0.03 | 0.05 | 0.01 | 0.00 |
| Usual activities | 0.31 | 0.25 | 0.14 | 0.30 | 0.23 | 0.09 | 0.12 | 0.15 | 0.20 | 0.11 | 0.16 | 0.11 |
| Pain/discomfort | 0.19 | 0.17 | 0.28 | 0.11 | 0.11 | 0.04 | 0.12 | 0.13 | 0.16 | 0.04 | 0.10 | 0.11 |
| Anxiety/depression | 0.16 | 0.15 | 0.10 | 0.19 | 0.19 | 0.08 | 0.06 | 0.08 | 0.19 | 0.04 | 0.13 | 0.09 |
| EQ-5D-3L utility | 0.33 | 0.27 | 0.20 | 0.28 | 0.29 | 0.14 | 0.11 | 0.26 | 0.13 | 0.08 | 0.19 | 0.15 |

CFQ-R-8D indicates Cystic Fibrosis Questionnaire–Revised–8 Dimensions.

^a^ Pearson correlations were used for continuous data; Spearman correlations were used for ordinal data (domains); weak <0.3; moderate <0.5 to ≥0.3; strong ≥0.5. Bold items – correlations as expected. Italicized – correlations not as expected.

# Supplementary Table 7. Pearson and Spearman rank correlation of change for SF-6D trials^a^

| **Change** | **Physical Functioning** | **Vitality** | **Emotional Functioning** | **Role Functioning** | **Respiratory Symptoms** | **Digestion** | **Body Image** | **Social Functioning** | **Health Perceptions** | **Weight** | **Eat** | **Treatment Burden** | **CFRSD** |
| --- | --- | --- | --- | --- | --- | --- | --- | --- | --- | --- | --- | --- | --- |
| CFQ-R-8D dimensions |  |  |  |  |  |  |  |  |  |  |  |  |  |
| Physical Functioning | **0.63** | 0.37 | 0.11 | 0.29 | 0.32 | -0.09 | 0.00 | 0.19 | 0.28 | 0.09 | 0.16 | 0.10 | 0.31 |
| Vitality | 0.34 | **0.61** | 0.20 | 0.27 | 0.25 | 0.06 | 0.08 | 0.20 | 0.23 | 0.12 | 0.19 | 0.20 | 0.34 |
| Emotional Functioning | 0.19 | 0.23 | **0.71** | 0.16 | 0.09 | 0.03 | 0.15 | 0.17 | 0.15 | 0.12 | 0.18 | 0.11 | 0.13 |
| Role Functioning | 0.26 | 0.30 | 0.09 | **0.64** | 0.20 | 0.15 | 0.07 | 0.18 | 0.26 | 0.07 | 0.11 | 0.06 | 0.24 |
| Cough | 0.36 | 0.27 | 0.06 | 0.26 | **0.54** | 0.07 | 0.07 | 0.10 | 0.32 | 0.20 | 0.14 | 0.09 | 0.50 |
| Breathing Difficulty | 0.36 | 0.25 | 0.04 | 0.25 | **0.78** | 0.06 | 0.00 | 0.21 | 0.27 | 0.18 | 0.14 | 0.09 | 0.40 |
| Abdominal Pain | 0.11 | 0.12 | -0.06 | 0.11 | 0.05 | **0.69** | 0.08 | 0.06 | 0.10 | 0.13 | 0.04 | 0.06 | 0.13 |
| Body Image | 0.09 | 0.02 | 0.21 | -0.03 | -0.03 | 0.11 | **0.52** | 0.05 | 0.07 | 0.11 | 0.06 | 0.02 | 0.05 |
| CFQ-R-8D utility | **0.63** | **0.56** | 0.38 | **0.58** | **0.61** | 0.31 | 0.18 | 0.32 | 0.46 | 0.25 | 0.28 | 0.21 | 0.55 |
|  |  |  |  |  |  |  |  |  |  |  |  |  |  |
| SF-6D dimensions |  |  |  |  |  |  |  |  |  |  |  |  |  |
| Physical functioning | **0.41** | 0.21 | 0.17 | 0.18 | 0.18 | 0.00 | 0.09 | 0.21 | 0.17 | 0.10 | 0.14 | 0.14 | 0.23 |
| Role limitation | 0.27 | 0.29 | 0.24 | **0.31** | 0.19 | 0.02 | 0.04 | 0.20 | 0.28 | 0.02 | 0.14 | 0.09 | 0.16 |
| Social functioning | 0.26 | 0.43 | 0.25 | 0.30 | 0.16 | -0.01 | 0.01 | 0.19 | 0.27 | 0.12 | 0.21 | 0.12 | 0.26 |
| Pain | 0.15 | 0.17 | 0.06 | 0.05 | 0.04 | *0.14* | 0.04 | -0.01 | 0.05 | 0.10 | 0.07 | -0.01 | 0.14 |
| Mental health | 0.15 | 0.20 | **0.41** | 0.10 | 0.09 | -0.02 | 0.10 | 0.17 | 0.10 | 0.11 | 0.14 | 0.09 | 0.12 |
| Energy/vitality | 0.39 | **0.44** | 0.26 | 0.34 | 0.20 | 0.01 | 0.05 | 0.31 | 0.34 | 0.17 | 0.25 | 0.10 | 0.28 |
| SF-6D utility | **0.37** | **0.43** | **0.40** | **0.40** | 0.20 | 0.06 | 0.10 | 0.31 | 0.41 | 0.08 | 0.23 | 0.13 | 0.27 |

CFQ-R-8D, Cystic Fibrosis Questionnaire–Revised–8 Dimensions; CFRSD Cystic Fibrosis Respiratory Symptom Diary; SF-6D, Short Form 6 dimensions.

^a^ Pearson correlations were used for continuous data; Spearman correlations were used for ordinal data (domains); weak <0.3; moderate <0.5 to ≥0.3; strong ≥0.5. Bold items – correlations as expected. Italicized – correlations not as expected.

# Supplementary Table 8. Responsiveness of generic and condition-specific measure from baseline to follow-up

| **Trial** | **EQ-5D trials**  **(n = 945)** | | | **SF-6D trial**  **(n = 405)** | | |
| --- | --- | --- | --- | --- | --- | --- |
| **Measure** | **Mean change (SD)**  **(T1-T0)** | **Effect size^a,b^** | **SRM^b,c^** | **Mean change (SD)**  **(T1-T0)** | **Effect size^a,b^** | **SRM^b,c^** |
| CFQ-R-8D | 0.004 (0.11) | 0.03 | 0.04 | 0.007 (0.10) | 0.06 | 0.07 |
| EQ-5D-3L | −0.001 (0.14) | −0.01 | −0.01 | – | – | – |
| SF-6D | **–** | **–** | **–** | 0.008 (0.11) | 0.07 | 0.08 |
| CFRSD | **–** | **–** | **–** | 1.259 (10.81) | 0.11 | 0.12 |

CFQ-R-8D indicates Cystic Fibrosis Questionnaire–Revised–8 Dimensions; CFRSD, Cystic Fibrosis Respiratory Symptom Diary; SF-6D, Short Form 6 dimensions;
SRM, standardized response mean; T0, baseline; T1, follow-up.

^a^ Effect size = mean change/baseline SD.

^b^ Small SRM/effect size ≥0.2 to <0.5; medium SRM/effect size ≥0.5 to <0.8; large SRM/effect size ≥0.8.

^c^ SRM = mean change/change SD.
